# Low relative hand grip strength is associated with a higher risk for diabetes and impaired fasting glucose among the Korean population

**DOI:** 10.1371/journal.pone.0275746

**Published:** 2022-10-06

**Authors:** Min Jin Lee, Ah Reum Khang, Dongwon Yi, Yang Ho Kang

**Affiliations:** 1 Division of Endocrinology and Metabolism, Department of Internal Medicine, Pusan National University Yangsan Hospital, Pusan National University School of Medicine, Yangsan, Korea; 2 Research Institute for Convergence of Biomedical Science and Technology, Pusan National University Yangsan Hospital, Yangsan, Korea; University of Perugia, ITALY

## Abstract

**Objective:**

This study investigated the association between relative hand grip strength (HGS) and glycemic status, such as impaired fasting glucose (IFG) and diabetes, using data from the Korea National Health and Nutrition Examination Survey (KNHANES).

**Methods:**

We performed a cross-sectional study using the data from the KNHANES of 27,894 individuals from 2014 to 2019. Relative HGS was defined as the absolute HGS divided by body mass index and divided into quartiles in men and women. Odds ratios (OR) for diabetes and IFG were calculated using multivariate logistic regression analysis. All analyses were stratified by sex, and subgroup analysis was age-stratified.

**Results:**

The lowest relative HGS quartile had a significant increase in the risk for diabetes (men: OR 2.72, 95% confidence interval [CI] 2.12–3.50; women: OR 3.38, 95% CI 2.70–4.24) and IFG (men: OR 1.35, 95% CI 1.15–1.59; women: OR 1.60, 95% CI 1.40–1.84). The ORs for diabetes and IFG according to the decreasing quartiles of relative HGS gradually increased in both sexes (*P* for trend <0.001). ORs and 95% CI of the lowest relative HGS quartile for diabetes were higher in the younger age group than that of the older age group (men: 4.47 and 2.80–7.14 for young adults; 2.41 and 1.37–4.25 for older adults; women: 5.91 and 3.06–9.38 for young adults; 1.47 and 0.92–2.33 for older adults). ORs and 95% CI for IFG was similar with the trend of ORs for diabetes (men: 1.80 and 1.43–2.26 for young adults; 1.17 and 0.75–1.84 for older adults; women: 2.20 and 1.77–2.72 for young adults; 1.33 and 0.86–2.07 for older adults).

**Conclusion:**

Lower relative HGS was associated with a higher risk of not only diabetes but also IFG in both sexes. These trends were stronger in younger adults than in older adults.

## Introduction

Insulin resistance is a preceding finding in individuals with prediabetes and type 2 diabetes mellitus (DM). Inflamed and dysfunctional adipocytes from the fat tissue is known to be the basis of development of insulin resistance during the initial stages of the disease, which simultaneously initiate or exacerbate insulin resistance in the muscle and liver [1]. The skeletal muscle is an important organ for glucose homeostasis and plays an important role in insulin-stimulated glucose uptake and disposal [2]. Insulin resistance in the skeletal muscle has important implications in the pathogenesis of type 2 DM and metabolic syndrome [3]. Low muscle mass is associated with insulin resistance and the incidence of type 2 DM after adjusting for fat mass [4,5]. In addition, low muscle quality is associated with diabetes and is an independent risk factor for poor glycemic control in individuals with type 2 DM [6–9].

Handgrip strength (HGS) is a simple and reliable tool for measuring the maximum voluntary force of the hands, and it is an indicator of the muscle quality that reflects muscle strength and muscle mass [10]. Low HGS is associated with adverse health outcomes, including chronic disease, nutritional status, frailty, and even mortality [11–14]. Therefore, recent consensus groups have accepted HGS as one of the criteria to diagnose sarcopenia, which is defined as the accelerated loss of muscle mass and function [10,15]. Previous studies have investigated the relationship between HGS and diabetes [16–21]. Recently, a meta-analysis of observational cohort studies suggested that HGS may be a risk indicator for type 2 DM [22]. However, as absolute grip strength has the limitation of not considering body size, relative HGS considering body size, that is, body mass index (BMI), can be a good alternative [23]. Epidemiological studies have shown that relative HGS is a useful indicator of type 2 DM, metabolic syndrome, and cardiovascular risk [24–28]. In addition, several studies have suggested that relative HGS is a better predictor than dominant or absolute HGS for new-onset diabetes and cardiometabolic risk [29–31].

Individuals with prediabetes have abnormal carbohydrate metabolism, but their glucose levels do not meet the criteria for diabetes, which is an important clinical entity in terms of risk factors for progression to diabetes and cardiovascular disease [32]. Recently, the prevalence of impaired fasting glucose (IFG), one of the criteria defining prediabetes, has increased to approximately 26.9% among the Korean population [33]. However, studies investigating the association between relative HGS and glycemic status, such as IFG and diabetes, are lacking. In the present study, we investigated whether relative HGS would be an indicator of glycemic status, not only diabetes, but also IFG, using data from the Korea National Health and Nutrition Examination Survey (KNHANES). As the relative HGS values were significantly different according to sex, we analyzed our data stratified by sex. Additionally, we conducted a subgroup analysis by age to find an age group that better reflects the relationship between relative HGS and glycemic status.

## Materials and methods

### Study population

**Fig 1:**
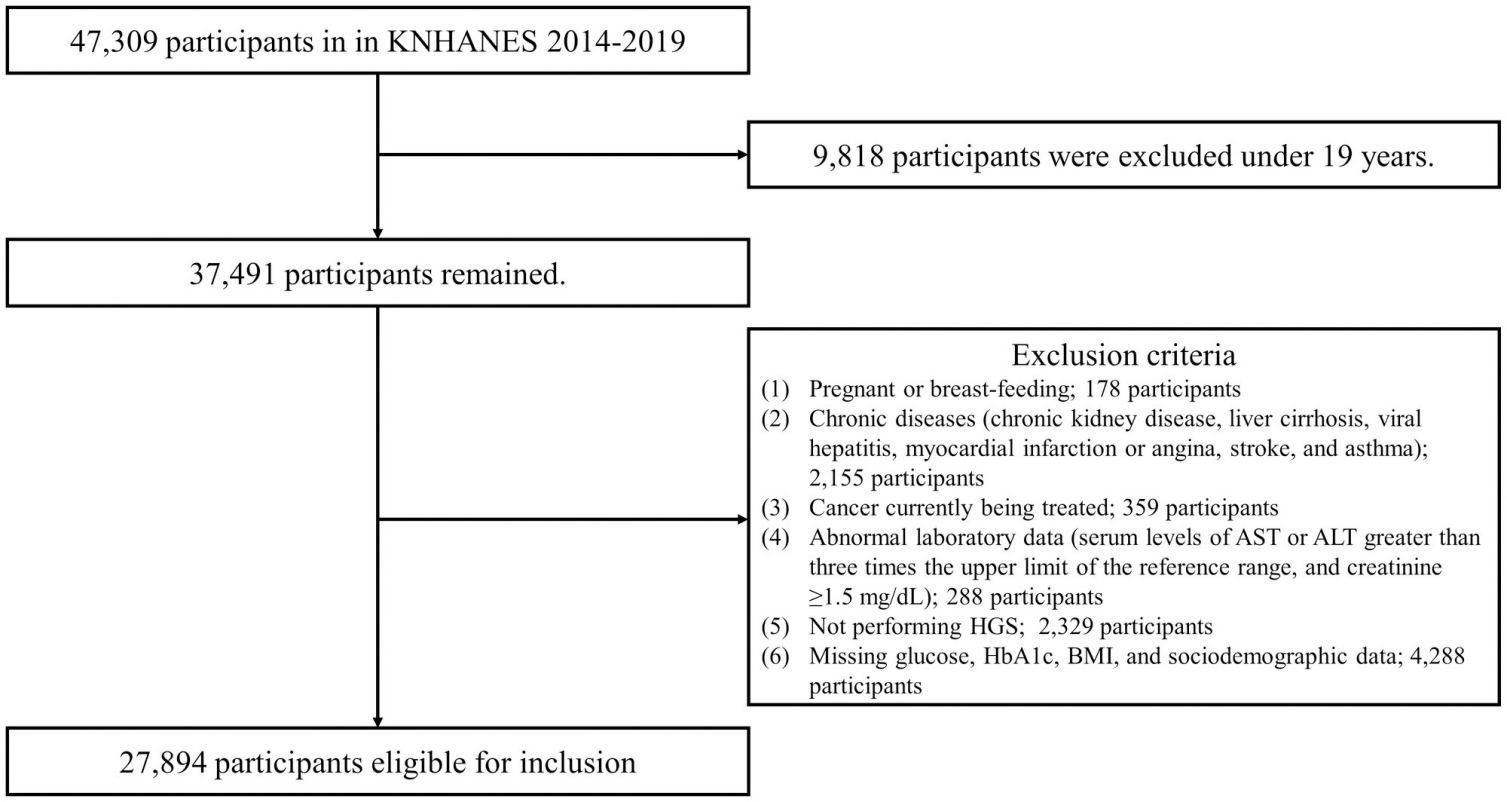
Flow diagram of the present study. KNHANES, Korea National Health and Nutrition Examination Survey; AST, aspartate aminotransferase; ALT, alanine aminotransferase; HGS, hand grip strength; BMI, body mass index.

The KNHANES is a nationwide cross-sectional survey performed annually using a stratified, multistage probability sampling method, and is representative of the general Korean population. We analyzed data from 2014 to 2019, which were included in the sixth, seventh, and eighth KNHANES, and when the HGS values were examined in these health examination surveys [34,35]. Of the 47,309 participants, 37,491 aged ≥20 years were examined. We excluded those who (1) were pregnant or breastfeeding; (2) had chronic diseases (such as chronic kidney disease, liver cirrhosis, viral hepatitis, myocardial infarction or angina, stroke, and asthma); (3) were undergoing cancer treatment; (4) had abnormal laboratory data (such as serum levels of aspartate aminotransferase or alanine aminotransferase >three times the upper limit of the reference range, and creatinine ≥1.5 mg/dL); (5) had not performed HGS (e.g., those with defects and injuries of the hand or fingers, a history of surgery of the hand or fingers in the last 3 months, and pain of the hand or fingers in the last 7 days); and (6) had missing glucose, HbA1c, BMI, and sociodemographic data; finally, 27,894 individuals were enrolled in the study (Fig 1).

The KNHANES was approved by the Institutional Review Board of the Korea Centers for Disease Control and Prevention, and all participants provided written informed consent. The study protocol was approved by the Institutional Review Board of the Pusan National University (IRB no. 05-2022-111).

### Data collection and measurement

Data from the KNHANES comprised health interviews, health examinations, and nutrition surveys. Sociodemographic factors and medical history were assessed through health interviews, including self-reported questionnaires, and personal interviews with trained staff, which included questions on household income level, education level, smoking status, amount of alcohol intake, physical activity, and past medical history. The smoking status was classified into three groups, i.e., never smokers, ex-smokers, or current smokers on the basis of lifetime smoking status. Never smokers were defined as those who had never smoked or had smoked <5 packs of cigarettes in their lifetime. Ex-smokers were defined as those who smoked ≥5 packs of cigarettes in their lifetime and were not presently smoking at the time of the survey. Current smokers were defined as those who had smoked ≥5 packs of cigarettes in their lifetime and were presently smoking at the time of the survey. Alcohol consumption status was classified into three groups: nondrinkers, light drinkers (<30 g/day), and moderate-to-heavy drinkers (≥30 g/day) on the basis of the amount of alcohol per day. Physically active subjects were defined as individuals who performed ≥150 min of moderate-intensity activity or ≥75 min of vigorous intensity activity per week. In the KNHANES data, household income was divided into sex- and age-specific quartiles of low, middle-low, middle-high, and high based on equalized household income, which was calculated by dividing the monthly household income by the square root of the number of household members. We recategorized the quartiles into tertiles by combining the middle-low and middle-high quartiles into the middle tertile.

Trained nurses performed anthropometric measurements. BMI was calculated as weight (kg) divided by height squared (m^2^). Waist circumference (WC) was measured at the midpoint between the lower margin of the last palpable rib and the top of the iliac crest during exhalation. Blood pressure (BP) was measured using a standard mercury sphygmomanometer after resting for 5 min in the sitting position. Systolic and diastolic BP were the average of the last two values among the three repetitive measures at 5-minute intervals.

All blood samples were obtained after an 8-hour fasting, and laboratory tests were conducted at a central laboratory within 24 h after sampling. Fasting plasma glucose (FPG), triglyceride, and high-density lipoprotein (HDL) cholesterol levels were measured using a Hitachi 7600 Automatic Analyzer (Hitachi, Tokyo, Japan). HbA1c levels were analyzed using G8 automated high-performance liquid chromatography (Tosoh, Tokyo, Japan).

### Measurement of handgrip strength

The KNHANES included HGS testing in 2014 to assess muscle strength [34]. A digital grip strength dynamometer (TKK 5401; Takei Scientific Instruments Co. Ltd., Tokyo, Japan) was used to measure HGS. The grip strength was assessed with the participants in a standing position, with the arms fully extended at the sides, and without bending the elbow or wrist. Trained staff instructed the participants to squeeze the dynamometer as firmly as they could, for <3 s, three attempts with each hand alternatively, with a 1-minute rest period. Absolute HGS (kg) was calculated as the summation of the maximal HGS value for each hand, and relative HGS (kg/BMI) was defined as the absolute HGS divided by BMI.

### Definitions of diabetes and IFG

Diabetes was defined as the presence of any of the following findings: FPG level ≥126 mg/dL, an HbA1c level ≥6.5%, self -reported physician’s diagnosis of diabetes, or current use of anti-diabetic medications [32]. IFG was defined as the presence of FPG levels of 100–125 mg/dL among individuals without diabetes [36].

### Statistical analysis

We performed a complex sample analysis considering the stratified multistage probability sampling design of the KNHANES. We used integrated weights from 2014 to 2019 for the analysis. The relative HGS was categorized into the following quartile groups: Q1 (lowest group), Q2, Q3, and Q4 (highest group). Because of sex-specific differences in relative HGS, all analyses were performed separately for men and women. The baseline characteristics of the participants were compared using the complex sample generalized linear model for continuous variables and complex sample crosstab analysis for categorical variables. Data are presented as weighted means or percentages with standard error (SE) for continuous and categorical variables, respectively. We used weighted linear regression analysis to determine an age-adjusted association between relative HGS and continuous variables. The odds ratios (ORs) and 95% confidence intervals (CI) for diabetes or IFG were calculated using the weighted logistic regression analysis. We used the rms package in R (version 3.6.3; R Core Team, Vienna, Austria) to draw the restricted cubic spline curve of the ORs for diabetes and IFG. In addition, we conducted subgroup analysis according to age (20–49, 50–64, and 65–80 years). A two-tailed P value of <0.05 was considered statistically significant. SPSS version 24 (IBM Corporation, Chicago, IL, USA) was used for all the calculations and analyses.

## Results

A total of 12,246 men and 15,648 women were enrolled in this study. The relative HGS for men were as follows: Q1, 0.459–2.808; Q2, 2.809–3.240; Q3, 3.241–3.686; and Q4, 3.687–6.312. For women, they were as follows: Q1, 0.406–1.680; Q2, 1.681–2.021; Q3, 2.022–2.361; and Q4, 2.362–4.129. The baseline characteristics of men and women according to relative HGS are presented in Tables 1 and 2, respectively. Both men and women with high relative HGS were more likely to be younger; have lower BMI, WC, systolic and diastolic BP, FPG, HbA1c level, and triglyceride level; and have higher HDL cholesterol level and absolute HGS. The group with highest relative HGS had a higher prevalence of current smokers, moderate-to-heavy drinkers, and physically active individuals of both sexes. The group with lowest relative HGS was more prevalent among both men and women with low economic and educational status. The age-adjusted linear regression coefficients between the relative HGS and clinical variables are shown in Table 3. In both sexes, BMI, WC, systolic and diastolic BP, FPG, HbA1c level, and triglyceride level showed a significant negative correlation with relative HGS (*P* <0.001), whereas HDL cholesterol level and absolute HGS showed a positive correlation with relative HGS (*P* <0.001).

**Table 1:** Baseline characteristics of men by quartiles of relative HGS.

Characteristics	Quartiles of relative HGS (n = 12,246)				*P* value
	Q1	Q2	Q3	Q4	
Range	0.459–2.808	2.809–3.240	3.241–3.686	3.687–6.312	
N	3,061	3,058	3,063	3,064	
Age (years)	51.19 (0.44)	46.27 (0.35)	44.37 (0.30)	39.88 (0.25)	**< .001**
BMI (kg/m^2^)	26.67 (0.09)	25.16 (0.07)	24.23 (0.06)	22.69 (0.06)	**< .001**
WC (cm)	92.12 (0.22)	87.88 (0.18)	85.34 (0.16)	80.92 (0.16)	**< .001**
SBP (mmHg)	122.66 (0.33)	120.57 (0.30)	119.28 (0.311)	116.33 (0.27)	**< .001**
DBP (mmHg)	78.57 (0.24)	79.00 (0.22) ^f^	78.85 (0.23)^g^	77.30 (0.20)	**< .001**
FPG (mg/dL)	107.00 (0.65)	103.35 (0.50)	100.94 (0.51)	96.31 (0.33)	**< .001**
HbA1c (%)	5.88 (0.02)	5.73 (0.02)	5.64 (0.02)	5.49 (0.01)	**< .001**
TG (mg/dL)^a^	167.08 (2.63)	174.98 (3.10)^h^	163.85 (2.81)	146.70 (3.17)	**< .001**
HDL-C (mg/dL)	44.81 (0.22)	46.89 (0.22)	47.99 (0.24)	50.55 (0.23)	**< .001**
Absolute HGS (kg)	64.72 (0.27)	76.36 (0.21)	83.72 (0.21)	92.92 (0.24)	**< .001**
Relative HGS (kg/BMI)^b^	2.43 (0.01)	3.04 (0.00)	3.46 (0.00)	4.11 (0.01)	**< .001**
Smoking status (%)					**< .001**
Never smoker	29.5 (1.0)	27.2 (1.0)	26.4 (0.9)	25.6 (0.9)	
Ex-smoker	40.7 (1.0)	37.4 (1.0)	34.9 (1.0)	31.3 (1.0)	
Current smoker	29.8 (1.0)	35.4 (1.0)	39.0 (1.1)	43.0 (1.1)	
Alcohol intake (%)					**< .001**
Nondrinker	21.1 (0.9)	13.7 (0.7)	12.0 (0.7)	11.0 (0.6)	
Light drinker	65.6 (1.1)	70.3 (1.0)	71.5 (0.9)	72.1 (0.9)	
Moderate-to-heavy drinker	13.2 (0.7)	16.0 (0.8)	16.6 (0.8)	16.9 (0.8)	
Physically active (%)					**< .001**
Yes	20.9 (0.9)	26.2 (1.0)	30.8 (1.0)	32.4 (1.0)	
No	79.1 (0.9)	73.8 (1.0)	69.2 (1.0)	67.6 (1.0)	
Household income (%)					**< .001**
Low	27.4 (1.1)	22.9 (1.0)	21.9 (1.0)	22.5 (0.9)	
Middle	48.0 (1.2)	51.0 (1.1)	52.3 (1.1)	53.0 (1.1)	
High	24.7 (1.1)	26.1 (1.0)	25.8 (1.0)	24.5 (1.0)	
Education level (%)					**< .001**
Elementary/middle school or less	26.2 (1.0)	17.0 (0.8)	13.4 (0.7)	8.0 (0.5)	
High school	33.2 (1.1)	35.1 (1.1)	37.9 (1.1)	43.5 (1.1)	
College or more	40.6 (1.2)	48.0 (1.2)	48.7 (1.2)	48.6 (1.2)	
Hypertension (%)^c^					**< .001**
Yes	42.0 (1.1)	32.6 (0.9)	24.3 (0.9)	16.5 (0.8)	
No	58.0 (1.1)	67.4 (0.9)	75.7 (0.9)	83.5 (0.8)	
Obesity status (%)^d^					**< .001**
Under and normal weight	15.0 (0.7)	23.3 (0.9)	31.8 (1.0)	54.3 (1.1)	
Overweight	20.1 (0.8)	25.0 (0.9)	30.3 (0.9)	26.6 (0.9)	
Obesity	64.9 (1.0)	51.7 (1.1)	37.9 (1.0)	19.1 (0.8)	
Glycemic status (%)^e^					**< .001**
Normal glucose	50.3 (1.1)	57.4 (1.1)	62.0 (1.0)	73.4 (0.9)	
IFG	30.0 (1.0)	29.4 (1.0)	28.4 (0.9)	21.9 (0.8)	
Diabetes	19.7 (0.8)	13.2 (0.7)	9.6 (0.6)	4.7 (0.4)	

Values are presented as weighted means (SE) for continuous or weighted percentages (SE) for categorical variables.

^a^ Logarithm-transformed values were used for analysis;

^b^ Defined as the absolute HGS divided by BMI;

^c^ Defined as a SBP of ≥140 mmHg, DBP of ≥90 mmHg, or use of antihypertensive medication;

^d^ Under and normal weight: <22.9 kg/m^2^, overweight: 23–24.9 kg/m^2^, obese: ≥25 kg/m^2^ using the World Health Organization Asian BMI cut points;

^e^ Diabetes: FPG level ≥126 mg/dL, an HbA1c level ≥6.5%, self -reported physician’s diagnosis of diabetes, or current use of anti-diabetic medications and IFG: FPG levels of 100–125 mg/dL among individuals without diabetes;

^f^ Q1 vs Q2, *P* = 0.146;

^g^ Q1 vs Q3, *P* = 0.402;

^h^ Q1 vs Q2, *P* = 0.635.

HGS, hand grip strength; BMI, body mass index; WC, Waist circumference; SBP, systolic blood pressure; DBP, diastolic blood pressure; FPG, fasting plasma glucose; TG, triglycerides; HDL-C, high density lipoprotein cholesterol; IFG, impaired fasting glucose; SE, standard error.

**Table 2:** Baseline characteristics of women by quartiles of relative HGS.

Characteristics	Quartiles of relative HGS (n = 15,648)				*P* value
	Q1	Q2	Q3	Q4	
Range	0.406–1.680	1.681–2.021	2.022–2.361	2.362–4.129	
N	3,911	3,910	3,915	3,912	
Age (years)	57.44 (0.38)	48.90 (0.34)	44.20 (0.28)	36.69 (0.22)	**< .001**
BMI (kg/m^2^)	26.01 (0.09)	24.02 (0.06)	22.57 (0.05)	20.91 (0.05)	**< .001**
WC (cm)	85.83 (0.22)	80.14 (0.17)	76.25 (0.16)	72.19 (0.15)	**< .001**
SBP (mmHg)	122.37 (0.38)	116.22 (0.31)	112.10 (0.28)	108.95 (0.25)	**< .001**
DBP (mmHg)	74.93 (0.23)	74.14 (0.18)	73.03 (0.18)	71.98 (0.18)	**< .001**
FPG (mg/dL)	104.10 (0.48)	98.03 (0.37)	94.88 (0.32)	91.88 (0.22)	**< .001**
HbA1c (%)	5.91 (0.02)	5.67 (0.01)	5.52 (0.01)	5.41 (0.01)	**< .001**
TG (mg/dL)^a^	133.90 (1.57)	116.81 (1.49)	103.56 (1.28)	59.90 (1.20)	**< .001**
HDL-C (mg/dL)	50.77 (0.22)	54.41 (0.24)	56.43 (0.23)	58.98 (0.25)	**< .001**
Absolute HGS (kg)	36.27 (0.16)	44.62 (0.12)	49.29 (0.12)	55.60 (0.13)	**< .001**
Relative HGS (kg/BMI)^b^	1.40 (0.01)	1.86 (0.01)	2.19 (0.01)	2.67 (0.01)	**< .001**
Smoking status (%)					**< .001**
Never smoker	90.6 (0.6)	88.7 (0.6)	88.3 (0.6)	85.8 (0.7)	
Ex-smoker	4.6 (0.4)	5.7 (0.5)	6.2 (0.4)	7.5 (0.5)	
Current smoker	4.8 (0.5)	5.5 (0.5)	5.5 (0.4)	6.7 (0.5)	
Alcohol intake (%)					**< .001**
Nondrinker	44.8 (1.0)	32.7 (0.9)	26.5 (0.8)	21.5 (0.8)	
Light drinker	50.4 (1.0)	60.6 (0.9)	67.1 (0.9)	71.3 (0.8)	
Moderate-to-heavy drinker	4.8 (0.5)	6.7 (0.5)	6.4 (0.5)	7.1 (0.5)	
Physically active (%)					**< .001**
Yes	9.1 (0.6)	15.7 (0.7)	18.9 (0.8)	23.4 (0.8)	
No	90.9 (0.6)	84.3 (0.7)	81.1 (0.8)	76.6 (0.8)	
Household income (%)					**< .001**
Low	30.3 (0.9)	24.5 (0.9)	21.8 (0.9)	22.2 (0.8)	
Middle	47.0 (0.9)	51.1 (1.0)	52.0 (1.0)	49.7 (1.0)	
High	22.7 (0.9)	24.4 (0.9)	26.2 (1.0)	28.0 (1.0)	
Education level (%)					**< .001**
Elementary/middle school or less	51.2 (1.1)	29.8 (0.9)	18.7 (0.7)	8.0 (0.5)	
High school	26.8 (0.9)	33.8 (0.9)	38.5 (1.0)	37.3 (1.0)	
College or more	22.0 (0.9)	36.4 (1.0)	42.8 (1.0)	54.7 (1.0)	
Hypertension (%)^c^					**< .001**
Yes	43.3 (1.0)	25.2 (0.8)	15.3 (0.6)	8.0 (0.5)	
No	56.7 (1.0)	74.8 (0.8)	84.7 (0.6)	92.0 (0.5)	
Obesity status (%)^d^					**< .001**
Under and normal weight	22.5 (0.8)	39.9 (0.9)	59.0 (0.9)	82.5 (0.7)	
Overweight	20.0 (0.7)	25.3 (0.8)	22.5 (0.8)	12.4 (0.6)	
Obesity	57.4 (1.0)	34.8 (0.9)	18.5 (0.7)	5.1 (0.4)	
Glycemic status (%)^e^					**< .001**
Normal glucose	55.4 (1.0)	70.8 (0.8)	77.1 (0.8)	86.0 (0.6)	
IFG	24.1 (0.8)	19.5 (0.7)	17.3 (0.7)	11.4 (0.5)	
Diabetes	20.5 (0.7)	9.7 (0.5)	5.6 (0.4)	2.6 (0.3)	

Values are presented as weighted means (SE) for continuous or weighted percentages (SE) for categorical variables.

^a^ Logarithm-transformed values were used for analysis;

^b^ Defined as the absolute HGS divided by BMI;

^c^ Defined as a SBP of ≥140 mmHg, DBP of ≥90 mmHg, or use of antihypertensive medication;

^d^ Under and normal weight: <22.9 kg/m^2^, overweight: 23–24.9 kg/m^2^, obese: ≥25 kg/m^2^ using the World Health Organization Asian BMI cut points;

^e^ Diabetes: FPG level ≥126 mg/dL, an HbA1c level ≥6.5%, self -reported physician’s diagnosis of diabetes, or current use of anti-diabetic medications and IFG: FPG levels of 100–125 mg/dL among individuals without diabetes.

HGS, hand grip strength; BMI, body mass index; WC, Waist circumference; SBP, systolic blood pressure; DBP, diastolic blood pressure; FPG, fasting plasma glucose; TG, triglycerides; HDL-C, high density lipoprotein cholesterol; IFG, impaired fasting glucose; SE, standard error.

**Table 3:** Age-adjusted regression coefficients for relative HGS by sex.

Variables	Men (n = 12,246)				Women (n = 15,648)			
	Beta	95% CI		*P* value	Beta	95% CI		*P* value
		Lower	Upper			Lower	Upper	
BMI	-0.090	-0.093	-0.086	**< .001**	-0.063	-0.065	-0.061	**< .001**
WC	-0.032	-0.033	-0.031	**< .001**	-0.021	-0.022	-0.021	**< .001**
SBP	-0.005	-0.006	-0.004	**< .001**	-0.003	-0.004	-0.003	**< .001**
DBP	-0.003	-0.005	-0.002	**< .001**	-0.003	-0.004	-0.002	**< .001**
FPG	-0.003	-0.003	-0.002	**< .001**	-0.003	-0.003	-0.003	**< .001**
HbA1c	-0.082	-0.098	-0.066	**< .001**	-0.092	-0.104	-0.079	**< .001**
TG^a^	-0.117	-0.139	-0.095	**< .001**	-0.15	-0.166	-0.135	**< .001**
HDL-C	0.011	0.010	0.012	**< .001**	0.006	0.005	0.007	**< .001**
Absolute HGS	0.033	0.032	0.034	**< .001**	0.038	0.037	0.038	**< .001**

^a^ Logarithm-transformed values were used for analysis.

HGS, hand grip strength; CI, confidence interval; BMI, body mass index; WC, Waist circumference; SBP, systolic blood pressure; DBP, diastolic blood pressure; FPG, fasting plasma glucose; TG, triglycerides; HDL-C, high density lipoprotein cholesterol.

**Fig 2:**
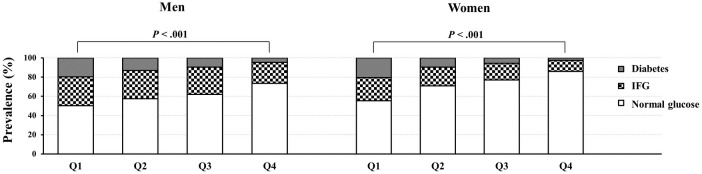
Prevalence of glycemic status according to the relative HGS quartiles. The relative HGS was categorized into the following quartile groups: Q1 (lowest group), Q2, Q3, and Q4 (highest group). IFG, impaired fasting glucose; HGS, hand grip strength.

**Fig 3:**
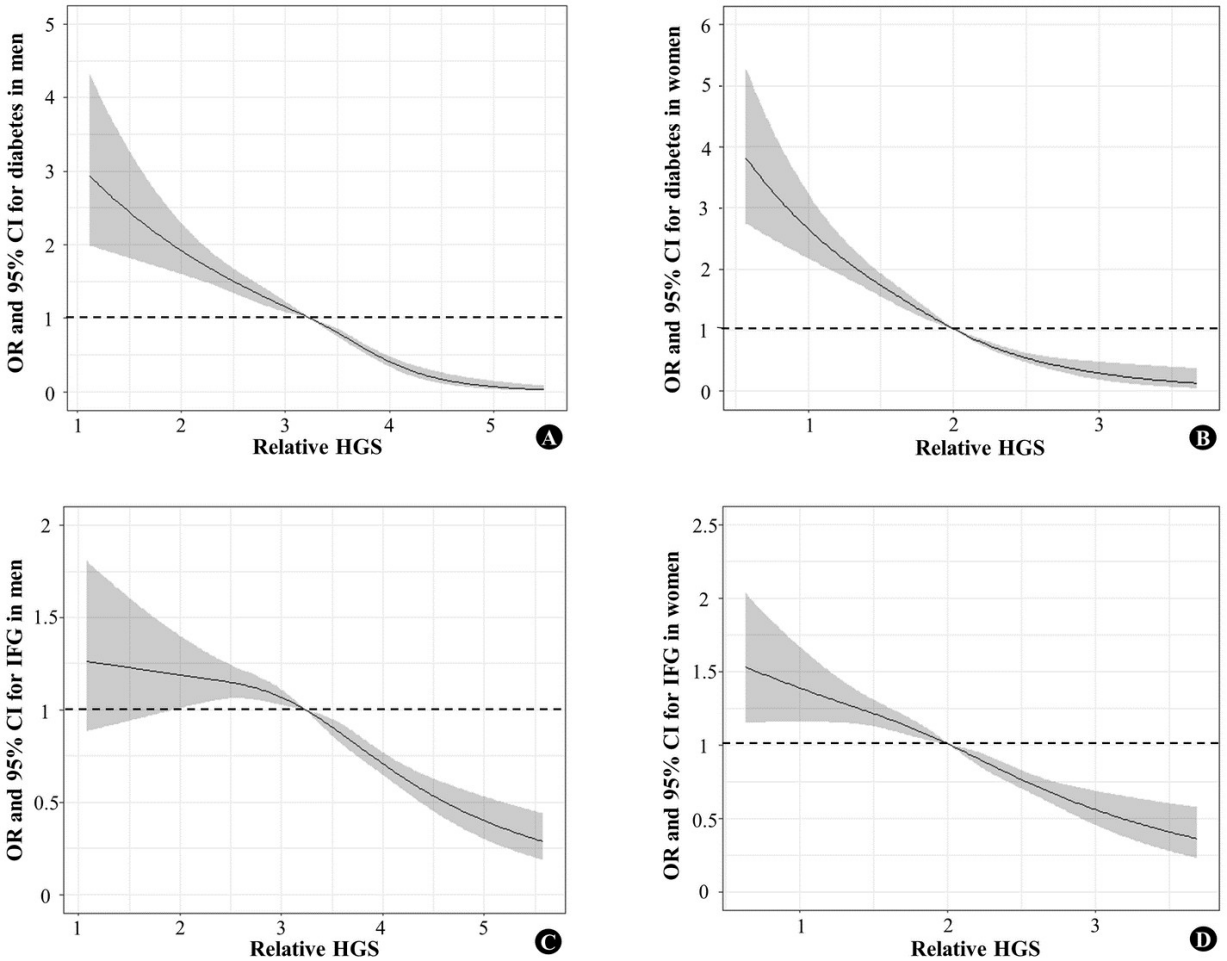
Restricted cubic spline models estimating ORs with 95% CIs of diabetes and IFG. (A) OR of diabetes in men. (B) OR of diabetes in women. (C) OR of IFG in men. (D) OR of IFG in women. The solid line represents the estimated OR of diabetes and IFG after adjustment; the shaded region represents the 95% CI. The reference value were the median of relative HGS (3.24 for men; 2.02 for women); the models were adjusted for age, SBP, TG, HDL-C, alcohol intake, smoking status, physically active, household income, and education levels. OR, odds ratio; CI, confidence interval; HGS, hand grip strength; IFG, impaired fasting glucose; SBP, systolic blood pressure; TG, triglycerides; HDL-C, high density lipoprotein cholesterol.

The prevalence of individuals with normal glucose, IFG, or diabetes according to relative HGS quartiles is presented in Fig 2. The group with the highest relative HGS showed normal glucose levels, 73.4%; IFG, 21.9%; and diabetes, 4.7% in men and normal glucose levels, 86.0%; IFG, 11.4%; and diabetes, 2.6% in women. The group with lowest relative HGS showed normal glucose levels, 50.3%; IFG, 30.0%; and diabetes, 19.7% in men and normal glucose, 55.4%; IFG, 24.1%; and diabetes, 20.5% in women. ORs and 95% CIs for diabetes and IFG according to decreasing quartiles, considering the highest relative HGS group as reference, are presented in Table 4. Individuals with the lowest relative HGS had a significant increase in the risk of diabetes in both men (OR, 2.72; 95% CI 2.12–3.50) and women (OR, 3.38; 95% CI 2.70–4.24). The ORs for diabetes according to the decreasing quartiles of relative HGS gradually increased in both sexes (*P* for trend <0.001). When the relative HGS value decreased by 1, the risk of diabetes was significantly increased in both men (OR, 1.78; 95% CI 1.57–2.02) and women (OR, 2.13; 95% CI 1.77–2.55). Considering the median of relative HGS (3.24 for men; 2.02 for women) as the reference value, as the relative HGS increased, the risk of diabetes decreased, and as the relative HGS decreased, the risk of diabetes increased in both sexes (Fig 3A and 3B).

**Table 4:** Adjusted ORs (95% CI) for diabetes and IFG according to quartiles of relative HGS by sex.

	Men			Women		
**Diabetes**	**OR (95% CI)**			**OR (95% CI)**		
	**Unadjusted**	**Model 1**	**Model 2**	**Unadjusted**	**Model 1**	**Model 2**
Q1	**6.12 (4.96–7.54)**	**2.91 (2.31–3.67)**	**2.72 (2.12–3.50)**	**12.28 (9.71–15.52)**	**4.01 (3.06–5.25)**	**3.38 (2.70–4.24)**
Q2	**3.59 (2.92–4.43)**	**2.30 (1.84–2.86)**	**2.20 (1.73–2.82)**	**4.57 (3.53–5.91)**	**2.42 (1.85–3.17)**	**2.11 (1.68–2.64)**
Q3	**2.43 (1.94–3.03)**	**1.79 (1.42–2.25)**	**1.80 (1.41–2.31)**	**2.40 (1.84–3.15)**	**1.77 (1.35–2.32)**	**1.62 (1.28–2.05)**
Q4	1 (reference)	1 (reference)	1 (reference)	1 (reference)	1 (reference)	1 (reference)
*P* for trend	**< .001**	**< .001**	**< .001**	**< .001**	**< .001**	**< .001**
Continuous^a^	**2.79 (2.53–3.08)**	**1.86 (1.66–2.09)**	**1.78 (1.57–2.02)**	**7.29 (6.35–8.34)**	**2.93 (2.45–3.49)**	**2.13 (1.77–2.55)**
**IFG**	**OR (95% CI)**			**OR (95% CI)**		
	**Unadjusted**	**Model 1**	**Model 2**	**Unadjusted**	**Model 1**	**Model 2**
Q1	**2.00 (1.75–2.30)**	**1.41 (1.22–1.64)**	**1.35 (1.15–1.59)**	**3.28 (2.84–3.77)**	**1.78 (1.52–2.09)**	**1.60 (1.40–1.84)**
Q2	**1.72 (1.50–1.96)**	**1.40 (1.22–1.61)**	**1.30 (1.12–1.51)**	**2.08 (1.80–2.40)**	**1.48 (1.28–1.72)**	**1.40 (1.23–1.60)**
Q3	**1.54 (1.35–1.75)**	**1.34 (1.18–1.53)**	**1.27(1.11–1.46)**	**1.69 (1.46–1.95)**	**1.43 (1.24–1.65)**	**1.37 (1.20–1.56)**
Q4	1 (reference)	1 (reference)	1 (reference)	1 (reference)	1 (reference)	1 (reference)
*P* for trend	**< .001**	**< .001**	**< .001**	**< .001**	**< .001**	**< .001**
Continuous^a^	**1.49 (1.38–1.61)**	**1.22 (1.13–1.32)**	**1.18 (1.09–1.30)**	**2.52 (2.28–2.79)**	**1.55 (1.39–1.74)**	**1.36 (1.20–1.54)**

Model 1was adjusted for age.

Model 2 was adjusted for age, SBP, TG, HDL-C, alcohol intake, smoking status, physically active, household income, and education levels.

^a^ Decrease by 1 of relative HGS value.

OR, odds ratio; CI, confidence interval; IFG, impaired fasting glucose; HGS, hand grip strength; SBP, systolic blood pressure; TG, triglycerides; HDL-C, high density lipoprotein cholesterol.

The trend of ORs of the relative HGS quartiles for IFG was similar to that of the ORs for diabetes in both sexes (Table 4). Individuals with the lowest relative HGS had a significant increase in the risk of IFG in both men (OR, 1.35; 95% CI 1.15–1.59) and women (OR, 1.60; 95% CI 1.40–1.84). Considering the median of relative HGS (3.24 for men; 2.02 for women) as the reference value, as the relative HGS increased, the risk of IFG decreased, and as the relative HGS decreased, the risk of IFG increased, similar to the spline curves of the ORs for diabetes (Fig 3C and 3D).

**Fig 4:**
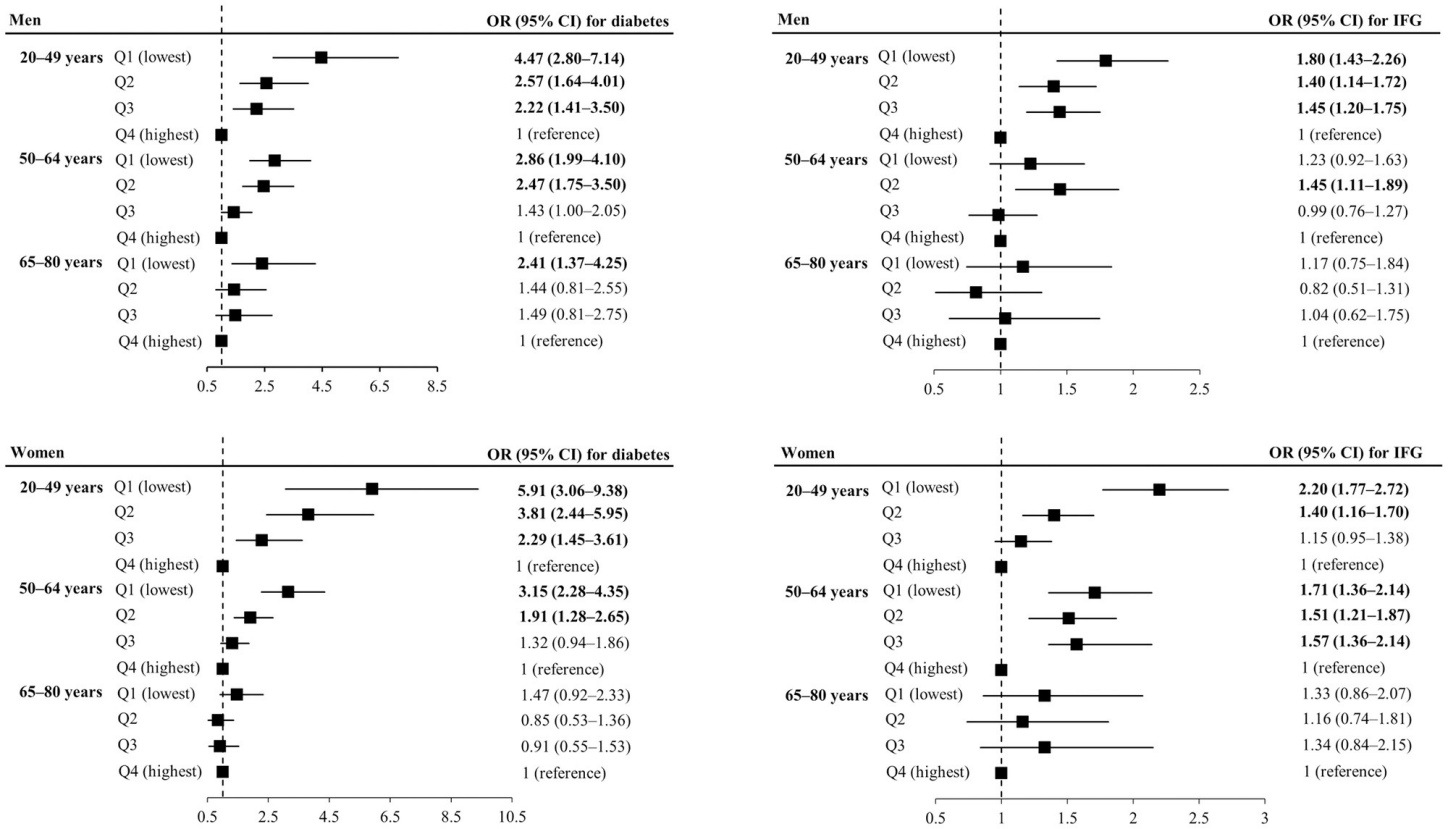
Odds ratios of diabetes and IFG in age-stratified subgroup analyses. Analyses were adjusted for age, SBP, TG, HDL-C, alcohol intake, smoking status, physically active, household income, and education levels. HGS, hand grip strength; IFG, impaired fasting glucose; SBP, systolic blood pressure; TG, triglycerides; HDL-C, high density lipoprotein cholesterol.

We further performed subgroup analysis according to age (20–49, 50–64, and 65–80 years) (Fig 4). Compared to the highest quartiles, the ORs and 95% CI of 1st–3rd quartiles for diabetes among individuals aged younger than 50 years (Q1, 4.47, and 2.79–7.14 for men; Q1, 5.91, and 3.06–9.38 for women), were higher than the ORs of those among individuals aged older than 65 years (Q1, 2.41, and 1.37–4.25 for men; Q1, 1.47 and 0.92–2.33 for women). In age-subgroup analyses, the ORs of the relative HGS quartiles for IFG were higher in the younger age group than in the older age group, which was similar to the trend of ORs for diabetes (Fig 4).

## Discussion

In this cross-sectional study, using the representative national data from KNHANES, from 2014 to 2019, we found that the groups with lower relative HGS reported higher ORs not only for diabetes but also for IFG in both the sexes. As the quartile of relative HGS decreased, the ORs for diabetes and IFG gradually increased for both the sexes. Additionally, considering the median relative HGS (3.24 for men; 2.02 for women) as the reference value, increased relative HGS was associated with a lower risk of DM and IFG, whereas decreased relative HGS was associated with a higher risk of DM and IFG. Furthermore, we observed that the relationship between relative HGS and glycemic status was more prominent in the younger age group than in the older age group.

A number of previous studies investigated the association of HGS, as a proxy for muscle strength with diabetes; however, the results have been conflicting. Some studies showed that HGS is inversely associated with type 2 DM [19,37–42] whereas other studies showed no association between HGS and type 2 DM [43,44]. Because relative HGS considers body size, i.e., BMI or weight as the denominator, relative HGS can be a useful indicator of insulin resistance and type 2 DM [24,29,45]. A cross-sectional study demonstrated that higher grip strength divided by body weight is associated with a lower prevalence of type 2 DM among Korean adults [24]. In a longitudinal study, relative HGS and absolute HGS divided by BMI predicted new-onset diabetes in the middle-aged and older European population [29]. Similarly, relative HGS considering body weight was inversely associated with the triglyceride glucose index and incidence risk of diabetes in older Chinese men [45]. In this study, we also demonstrated that the lowest relative HGS was significantly associated with an increased risk of diabetes in both men (OR 2.72) and women (OR 3.76).

Grip strength is inversely associated with serum glucose levels in a linear regression model in several studies [8,17,19,46]. In addition, the relative HGS was inversely associated with the triglyceride glucose index, reflecting insulin resistance [45]. We also demonstrated a linear inverse correlation between relative HGS, FPG, and HbA1c levels after adjusting for age. Transforming the glucose level of continuous variables to the glycemic status of categorical variables, such as normal glucose, prediabetes, and diabetes, revealed an inverse relationship between the relative HGS values and the prevalence of prediabetes. Hu et al. suggested that increased grip strength per body weight was associated with a lower prevalence of prediabetes among Chinese adults [47]. Although some differences in the definition of relative HGS and prediabetes existed compared to our study, their results are consistent with our study showing that the grip strength is a useful indicator for prediabetes in both the sexes. In another Korean population-based study using data from the KNHANES, Jang et al. showed that groups with low relative HGS had increased odds of prediabetes in men but not in women [48]. Compared with our study, the different results in women might be due to the difference in the definition of prediabetes. Jang et al. defined prediabetes using HbA1c cut-off of 5.7–6.4%, whereas we defined IFG using FPG levels of 100–125 mg/dL. A study using KNHANES data showed that the HbA1c criterion markedly increased the prevalence of prediabetes in women compared to that in men [36]. We thought that the non-significant result associated with female subjects in the study by Jang et al. was caused by the inclusion of participants with lower glucose levels than those of our study belonging to the prediabetes group. In a study investigating the association between relative HGS and IFG, similar to our study, a higher relative HGS was associated with a decreased risk of IFG in both the sexes [49]. In addition, a prospective cohort study in Japan demonstrated that a higher relative HGS predicted prediabetes incidence after 2 years of follow up [50]. In this study, we investigated the association between relative HGS and glycemic status, such IFG and diabetes, and demonstrated that a lower relative HGS was associated with a higher risk of both diabetes and IFG in both the sexes. The OR for diabetes was more prominent than the OR for IFG in the groups with low HGS, which is a natural result because of the larger glucose difference between normal glucose status and diabetes than between normal glucose status and IFG.

The pathophysiological mechanism underlying the relationship between muscle strength and glycemic status is not fully understood, but several explanations have been suggested. Increased skeletal muscle strength because of exercise training contributes to improved insulin action, glucose disposal, and enhanced muscle glycogen storage by increasing skeletal muscle GLUT4 expression [51]. Skeletal muscle fat infiltration, known as myosteatosis induced by aging, sedentary lifestyle, genetic predisposition, and unknown causes, is related to skeletal muscle function deficit and disruption of metabolism, and is greater in those with DM [52]. Inversely, hyperglycemia and insulin resistance in DM impair mitochondrial function in the muscles and lead to skeletal muscle insulin resistance, which is related to attenuated muscle strength [53]. On the basis of these mechanisms, previous studies verified that HGS, a feasible tool reflecting muscle strength, is inversely associated with type 2 DM [16,18,19,37–41]. Moreover, a meta-analysis of observational cohort studies suggested that HGS might be a risk indicator for type 2 DM [22]. Several epidemiological studies have verified the association between low muscle quality and poor glycemic control in patients with diabetes [8,17]. We also demonstrated an inverse relationship between the muscle strength calculated using relative HGS and the glycemic status categorized by normal glucose, IFG, and diabetes. As the relative HGS was calculated by dividing absolute HGS by BMI, we can carefully suggest that both, increasing HGS and decreasing BMI, intensify the protective effect of IFG as well as diabetes.

The use of allometric scales for removing the body size effect in HGS has been proposed, and Neto et al. suggested that body height is the best body size variable for normalization of HGS among older adults [54]. Recently, the Sarcopenia Definition and Outcomes Consortium presented a set of position statements of the sarcopenia definition, in which grip strength, either absolute or scaled to measures of body size (i.e., BMI, weight, total body fat, lean mass, and body weight), was an important indicator of slowness and low grip strength, with or without normalization to weight or BMI, was an indicator of adverse health outcomes [55]. We chose the BMI scale for normalization of HGS because BMI is a commonly used index to diagnose obesity and can be used to evaluate the risks of obesity-associated diseases, such as type 2 DM, hypertension, dyslipidemia, and cardiovascular diseases [56]. Several studies, similar to our study, used HGS divided by the BMI to investigate the relationship with metabolic syndrome, diabetes, and cardiovascular risk [24–28]. Other studies have suggested that HGS divided by the BMI is a better predictor of new-onset diabetes and cardiometabolic risk than dominant or absolute HGS [29–31]. Further studies are needed to investigate which allometric scale for estimating the normalization of HGS is best for reflecting metabolic parameters.

In this study, we showed that groups with a lower relative HGS were associated with a higher risk of diabetes and IFG in both men and women. Furthermore, we conducted a subgroup analysis stratified by age, which is the most important factor influencing glycemic status and HGS. Chun et al. investigated grip strength measures according to age group in Koreans [57]. They showed that the values of normalized HGS, such as relative HGS in our study, were the highest in the 35–40 years group and gradually decreased in the older age groups, slightly aggravated in the gradient beyond age 60–65 years in men, and steepening starting from age 50 to 55 years in women [57]. Considering the change in relative HGS by age in Koreans, we stratified age groups into 20–49, 50–64, and 65–80 years and showed that the inverse relationship between relative HGS and glycemic status was prominent among 20–50 years and non-significant among 65–80 years. In a cohort study, the relationship between relative muscle mass (i.e., total skeletal muscle mass per body weight) and the risk of incident type 2 DM was stronger in young and middle-aged adults (<50 years) [4]. In a UK biobank prospective cohort study, the association between low grip strength per body weight and the risk of type 2 DM incidence was stronger in individuals aged <55 years [20]. A Japanese study on the relationship between relative HGS and prediabetes incidence showed a stronger inverse relationship in young adults (<40 years) than in older adults [48]. In addition, Jang et al. showed that groups with lower relative HGS had significantly increased odds of prediabetes in men aged 30–39 and 50–59 years [50]. These studies, including ours, suggest that relative grip strength, as a representative of muscle quality, could be an important factor in ameliorating glycemic status, especially in young adults. Further pathophysiological studies are warranted to clarify the relationship between relative HGS and glycemic status in young adults.

This study had certain limitations. First, we used cross-sectional data, and therefore could not clarify the causal relationship. Although we demonstrated that the FPG and HbA1c levels had a significant inverse correlation with relative HGS, we could not exclude the effect of antidiabetic agents on grip strength in patients with diabetes. Second, we could not evaluate the glycemic status of impaired glucose tolerance because the KNHANES conducted between 2014 and 2019 did not investigate the 75 g oral glucose tolerance test. Although prediabetes can be defined by FPG, 2-hour glucose levels after a 75 g oral glucose tolerance test, and HbA1c [32]; we chose IFG defined by FPG as the status of prediabetes because the FPG level is a more widely used and inexpensive measure to evaluate glycemic status in individuals with or without diabetes, and a few discordances exist between FPG and HbA1c for defining prediabetes in people with different characteristics [36]. Third, as the KNHANES did not distinguish the type of diabetes, we defined diabetes without classifying the type of diabetes. However, as the prevalence of type 1 DM was approximately 0.017% to 0.021% of the entire population of Korea in the National Health Insurance Database [58], most participants with diabetes in our study had type 2 DM. Fourth, we analyzed the KNHANES data from 2014 to 2019, when the HGS values were examined but the body composition data were not. Therefore, we could not investigate the absolute amounts of skeletal muscle and visceral fat or their ratio. In addition, the health interviews conducted by the KNHANES did not include the status of neurologic deficit, malnutrition, or cachexia, which affect muscle quality. Instead, we excluded those with chronic diseases and undergoing cancer treatment, who were likely to have malnutrition or cachexia (Fig 1). The KNHANES data did not include the history of drugs, such as steroids, which influence insulin sensitivity, and we could not exclude the effect of steroids on the relationship between grip strength and glycemic status. Fifth, we used the national population-based data; therefore, our results are not applicable to other ethnic groups. Despite these limitations, the strength of this study is that it is the first large-scale study to show an inverse relationship between relative HGS values and the risk of IFG and diabetes. In addition, we systematically analyzed these relationships on the basis of sex, adjusted for several relevant variables, and conducted age-stratified subgroup analysis. This study used a nationally representative sample and showed the clinical importance of high relative HGS, i.e., increasing HGS or decreasing BMI, in the Korean population.

In conclusion, a lower relative HGS was associated with a higher risk of not only diabetes but also IFG in both the sexes. In addition, this relationship was more prominent in the younger age group. On the basis of our study findings, we suggest that both, increasing HGS and decreasing BMI, intensify the protective effect of IFG and diabetes, and this protective effect is more pronounced in young adults than in older adults. Further studies are required to clarify the causal relationship and the pathophysiology underlying this relationship.
